# Parallel cortical-brainstem pathways to attentional analgesia

**DOI:** 10.1016/j.neuroimage.2020.117548

**Published:** 2021-02-01

**Authors:** Valeria Oliva, Rob Gregory, Wendy-Elizabeth Davies, Lee Harrison, Rosalyn Moran, Anthony E. Pickering, Jonathan C.W. Brooks

**Affiliations:** a School of Physiology, Pharmacology and Neuroscience, Biomedical Sciences Building, University of Bristol, Bristol BS8 1TD, United Kingdom; b Anaesthesia, Pain and Critical Care Sciences, Bristol Medical School, University Hospitals Bristol, Bristol BS2 8HW, United Kingdom; c School of Psychological Science, University of Bristol, 12a Priory Road, Bristol BS8 1TU, United Kingdom; d Department of Neuroimaging, Institute of Psychiatry, Psychology & Neuroscience, King's College London, SE5 8AF, United Kingdom

**Keywords:** Pain, fMRI, Attention, Brainstem, Analgesia, Connectivity

## Abstract

Pain demands attention, yet pain can be reduced by focusing attention elsewhere. The neural processes involved in this robust psychophysical phenomenon, attentional analgesia, are still being defined. Our previous fMRI study linked activity in the brainstem triad of locus coeruleus (LC), rostral ventromedial medulla (RVM) and periaqueductal grey (PAG) with attentional analgesia. Here we identify and model the functional interactions between these regions and the cortex in healthy human subjects (*n* = 57), who received painful thermal stimuli whilst simultaneously performing a visual attention task. RVM activity encoded pain intensity while contralateral LC activity correlated with attentional analgesia. Psycho-Physiological Interaction analysis and Dynamic Causal Modelling identified two parallel paths between forebrain and brainstem. These connections are modulated by attentional demand: a bidirectional anterior cingulate cortex (ACC) – right-LC loop, and a top-down influence of task on ACC-PAG-RVM. By recruiting discrete brainstem circuits, the ACC is able to modulate nociceptive input to reduce pain in situations of conflicting attentional demand.

## Introduction

Attentional analgesia is a well-characterised phenomenon whereby increased cognitive load can decrease pain perception (Peyron et al., 2000; Bantick et al., 2002; Brooks et al., 2002; Valet et al., 2004; Brooks et al., 2017; Sprenger et al., 2012). This can be achieved by diverting attention from a painful stimulus to a visual task or simply by active mind-wandering (Bushnell et al., 2013; Kucyi et al., 2013). Central to attentional analgesia is the concept of divided attention, whereby less cognitive resource is available to be allocated to nociception and pain. Since noxious stimuli are inherently salient and therefore attention grabbing (Eccleston and Crombez, 1999), then any concurrent cognitive task must compete for ‘attentional’ resource. Attention is thus cast both as a key component of pain behaviour (i.e. attending to pain Crombez et al., 2004; Legrain et al., 2009; Roelofs et al., 2002) as well as a putative mechanism for pain relief. The processes regulating attentional focus is of importance in the development, maintenance and potentially resolution of chronic pain states.

The mechanisms that allow attention to regulate pain are currently not well understood and there has been ongoing debate about whether attentional analgesia requires engagement of descending control to attenuate nociception (Brooks et al., 2017; Bushnell et al., 2013; Lorenz et al., 2003; Tracey et al., 2002; Valet et al., 2004). These studies have linked several cortical regions to the attentional analgesic effects, including the anterior cingulate cortex (ACC), dorsolateral prefrontal cortex (dlPFC) and also components of the descending pain control system including periaqueductal grey (PAG), rostroventromedial medulla (RVM) and locus coeruleus (LC). An interaction between cortical and mid-brain structures during distraction from pain has been identified (Lorenz et al., 2003; Valet et al., 2004), but these previous studies were unable to examine interactions between the pontomedullary regions that are known to be important for the descending control of nociception.

The PAG, RVM and LC are all candidates for mediating attentional analgesia given their known anti-nociceptive roles (Millan, 2002). For example, multiple animal studies have demonstrated that interactions between the PAG and RVM produces endogenous analgesia, mediated by spinally projecting neurons in the RVM (Basbaum and Fields, 1979; Fields and Basbaum, 1978; Heinricher et al., 2009). Together with the ACC, these regions form one of the main pain modulatory pathways involved in the bidirectional modulation (i.e. facilitation and inhibition) of nociception in the spinal cord dorsal horn (De Felice and Ossipov, 2016; Ossipov et al., 2010; Quintero, 2013).

Similarly, the LC is another potential candidate region that could mediate the interaction between attention and pain because of its projections to the spinal cord which release noradrenaline to produce analgesia (Hirschberg et al., 2017; Llorca-Torralba et al., 2016). Additionally, it has a known role in salience signalling and attention mediated by ascending projections (Aston-Jones et al., 1999; Sales et al., 2019; Sara and Bouret, 2012). Despite it being challenging to resolve with fMRI (Astafiev et al., 2010; Liu et al., 2017), the LC was recently identified as the only region whose activity reflected the interaction between task and temperature in an attentional analgesia paradigm (Brooks et al., 2017). The LC could therefore contribute to attentional analgesia as part of the PAG-RVM system, or as a parallel descending modulatory pathway perhaps receiving inputs directly from ACC (Aston-Jones et al., 1991; Bajic and Proudfit, 1999).

Within this framework the ACC is ideally placed to mediate between competing cognitive demands (e.g. between a sustained visual attention task and pain) as it is active during conflict resolution (Braver et al., 2001; Kerns, 2006; Kim et al., 2011), its activity is modulated by attention (Davis et al., 2000) as well as being consistently activated by painful stimuli (Brooks et al., 2017; Garcia-Larrea and Peyron, 2013; Peyron et al., 2000; Wager et al., 2013). The ACC is known to code for pain intensity (Büchel et al., 2002; Coghill et al., 2003) and unpleasantness (Rainville et al., 1997), furthermore, sub-divisions (e.g. dorsal anterior ACC) are involved in high level cognitive appraisal of pain, including attention (Büchel et al., 2002). Some have proposed a specific role for dorsal ACC (dACC) in pain perception (Lieberman and Eisenberger, 2015), though this is disputed with other studies suggesting that activity within this structure reflects the multifaceted nature of pain (Wager et al., 2016). Connectivity between the ACC and structures involved in descending pain control e.g. the PAG, has been shown to vary with pain perception due to both attentional modulation of pain and placebo analgesic responses (Bantick et al., 2002; Eippert et al., 2009; Petrovic et al., 2000; Valet et al., 2004) suggesting a potential role in attentional analgesia.

We hypothesised a top-down pathway mediating attentional analgesia where the PAG receives attentional-shift signals from the ACC and/or LC and directs the RVM and/or LC to attenuate nociceptive processing in the spinal cord. Given the multiplicity of possible pathways and interactions by which activity in the brainstem can generate analgesia, we anticipated that effective connectivity analyses could resolve the roles of these regions (identified in our previous investigation (Brooks et al., 2017) during attentional analgesia. To increase the statistical power to undertake this connectivity analysis, additional fMRI datasets were acquired using the same paradigm as per Brooks et al. (2017). Analysis of these additional datasets reproduced our previous regional activation results, and so the three datasets were pooled for the effective connectivity analyses and modelling. We tested for psycho-physiological interactions (PPI, Friston et al., 1997; McLaren et al., 2012; O'Reilly et al., 2012) to explore whether the connectivity between the PAG, RVM, LC and ACC altered during the experimental paradigm. Finally, we used dynamic causal modelling (DCM, Friston et al., 2003) to test the directionality and strength of the connections.

## Methods

### Participants

Subjects were recruited using poster and email adverts at the University of Bristol for three different pain imaging studies at the Clinical Research and Imaging Centre (CRiCBristol) that used the same experimental paradigm: an initial study on attentional analgesia (Brooks et al., 2017), a study on sleep disruption and a study on fibromyalgia. The first two studies were approved by the University Bristol, Faculty of Science, Human Research Ethics Committee (reference 280,612,567 and 291,112,606 respectively) and the fibromyalgia study was approved by NHS South Central Oxford B Research Ethics Committee (reference 13/SC/0617).

All subjects gave written informed consent after application of standard inclusion/exclusion criteria for participation in MRI studies. The presence of significant medical/psychiatric disorders (including depression) or pregnancy precluded participation. Subjects with a chronic pain condition, or those who were regularly taking analgesics or psychoactive medications, as determined by self-report, were also excluded. All subjects were right-handed, verified with the Edinburgh handedness inventory (Oldfield, 1971).

The discovery cohort were 20 right-handed healthy subjects (median age 25 years, range 18–51 years, 10 females). Subjects attended for two sessions. During the screening visit, written consent was obtained and both task difficulty and temperature of the thermal stimulation were individually calibrated. Subsequently the subjects returned for the test session where they completed the experiment in the MRI scanner (For full details on the discovery cohort see Brooks et al. (2017).

The validation cohort composed of control subjects from two separate studies:

Twenty healthy volunteers (median age 23, range 20–33, 10 females) were recruited for a study investigating the effects of sleep disturbance on attentional analgesia. Subjects completed the same experiment protocol on two occasions; after a habitual and a disturbed night's sleep (at the sleep laboratory at CRiCBristol). For the present study, only data obtained from the control condition was used, wherein subjects experienced their habitual sleep regime the night prior to their scan. A second group of 20 healthy participants (median age 31.5, range 20–59, 18 females) was recruited from the control group of a study analysing attentional analgesia in fibromyalgia patients.

### Experiment

Thermal stimuli were delivered to the left volar forearm (approximately C6 dermatome) using a circular contact thermode (CHEPS Pathway, MEDOC) and each lasted 30 s. The noxious thermal stimulus was individually titrated to obtain a 6 out of 10 pain rating (42–45°C plateau). The innocuous stimulus plateau was set at 36°C. In both cases brief heat spikes of 2, 3 and 4°C above the plateau temperature were added in a random sequence at a frequency of 1 Hz. This heating profile was used to maintain painful perception, whilst avoiding skin sensitisation. The baseline thermode temperature was 32°C.

For the Rapid Serial Visual Presentation task (RSVP, Potter and Levy, 1969), subjects identified a visual target (the number “5″) among distractors (other letters and numbers), presented using back-projection to a screen visible to subjects lying in the scanner, responding with a button box (Lumina LP-400, Cedrus). Prior to entering the scanner, the speed of character presentation for the hard RSVP task was individually calibrated to obtain a 70% task performance. Task performance was assessed by calculating d’, a measure of task performance typically used in behavioural studies calculated by subtracting the z-transformation of the false alarm rate from the z-transformation of the hit rate (Stanislaw and Todorov, 1999). The d’ values were generated for a range of trial RSVP speeds for each subject and the data was fitted with a sigmoidal function (commonly used in psychophysics). This best fit model parameters were used to estimate each subject's presentation speed corresponding to a 70% task performance, which ranged from 32 to 96 ms. The speed of presentation for the easy RSVP task was either 192 or 256 ms, depending on performance in the hard task (if the “hard” task interval for the subject was <80 ms or >80 ms, respectively).

### Data acquisition

In the scanner, participants received noxious or innocuous thermal stimuli (high/low) while simultaneously performing the RSVP task with two levels of difficulty (easy/hard). Thus, there were four experimental conditions (in a 2 × 2 factorial experimental design): easy|low, easy|high, hard|low, hard|high. Each condition was repeated 4 times. Each experimental epoch started with instructions (5 s), followed by the 30 s experimental condition, followed by a 10 s rest period before an 8 s rating period where subjects rated the perceived pain intensity from 0 to 10 on a visual analogue scale (VAS) (See Fig 1 in Brooks et al., 2017). The post-stimulus interval, between the rating period and subsequent instructions, was 17 s.

**Fig. 1:**
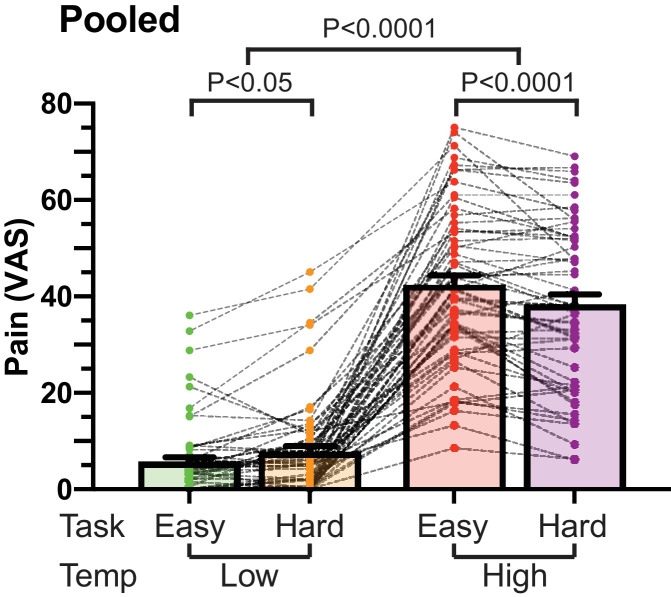
*Pain ratings across experimental conditions for the pooled cohort (N* *=* *57). A 2-way repeated measures ANOVA on the pain ratings showed the expected main effect of temperature (P < 0.0001) and a task x temperature interaction (P < 0.0001). The attentional analgesic effect was observed as a decrease in pain scores in the high temperature condition during the hard task compared to the easy task (P < 0.0001, post-hoc paired t-test). In contrast there was also a small increase in pain scores in the low temperature condition during the hard task compared to the easy task (P < 0.05). The main effect of task was not significant (P = 0.92). Error bars represent the standard error of the mean.*

The experiment for the validation cohort (*n* = 38) was essentially identical to that of the discovery cohort. The titrated mean high temperature for the discovery cohort was 44.2°C and for the validation cohort it was 43 °C (range 42–45 °C). The whole imaging session lasted 26 min for the discovery cohort and sleep-disruption cohort and was 22 min for the fibromyalgia cohort. The difference in experiment duration stemmed from the removal of a superfluous additional control condition, with no distraction during high temperature, in the fibromyalgia study as it was not required as part of the core 2 × 2 factorial design and had the additional benefit of reducing the number of noxious stimuli overall delivered to these subjects (and more importantly to the patients in the matched study group).

All data were acquired with a 3T Skyra MR system (Siemens Medical Solutions, Erlangen, Germany) and 32-channel receive only-head coil. In addition to blood oxygenation level dependent (BOLD) functional data, T1 weighted structural scans were acquired with an MPRAGE sequence to allow image registration. Functional imaging data were acquired with TE/TR=30/3000 ms, GRAPPA acceleration factor = 2, resolution = 1.5 × 1.5 × 3.5 mm. The slices were angulated perpendicularly to the base of the 4th ventricle to better match the orientation (long axis) of brainstem nuclei. This slice orientation optimised the ability to discriminate between the small brainstem structures in the transverse plane and while allowing the capture of whole brain activity within 3 s. Fieldmap data were acquired with a gradient echo sequence (TE1/TE2/TR = 4.92 / 7.38 / 520 ms, flip angle 60°, resolution 3 × 3 × 3 mm). During scanning, a pulse oximeter and a respiratory bellows (Expression MRI Monitoring System, InVivo, Gainesville, FL) were used to monitor cardiac pulse waveform and respiratory movement, recorded using an MP150 data logger (Biopac, Goleta, CA, USA) for subsequent physiological noise correction (Brooks et al., 2013).

### Behavioural data analysis

Pain VAS ratings were converted to a 0–100 scale for a repeated measures ANOVA in SPSS software (after Brooks et al., 2017). Following estimation of main effects (task, temperature) and interactions, post-hoc paired t-tests were performed. The presence of attentional analgesia was pre-defined as a significant interaction between task difficulty and high temperature on pain rating assessed with post-hoc paired t-testing (*p* < 0.05). To test for differences between the discovery and validation cohorts; group membership was added as a between subject factor to the two within subject factors (task and temperature). Subsequent analysis is reported on the pooled cohort.

### Imaging data analysis

#### Image pre-processing

Functional images were corrected for motion using MCFLIRT (Jenkinson et al., 2012) and co-registered to each subject's structural scan using brain boundary-based registration (Greve and Fischl, 2009) and then to the 2 mm template (“MNI152”) brain using a combination of fieldmap based unwarping using FUGUE (Jenkinson, 2003), linear transformation using FLIRT (Jenkinson and Smith, 2001) and non-linear registration using FNIRT (Andersson et al., 2007) with 5 mm warp spacing. Functional data were spatially smoothed with a kernel size of 3 mm (FWHM) and high pass temporally filtered with a 90 s cut-off. Two subjects in the validation cohort and one from the discovery cohort were excluded from the analyses at this stage because of signal dropout (primarily in the brainstem) in the EPI data, leaving 57 subjects.

Physiological data (cardiac and respiratory) were visually inspected and manually corrected as required. All first level models (block design and gPPI) included a basis set of regressors for physiological noise correction, which included 16 cardiac and respiratory terms (sine and cosine terms up to the 4th harmonics), plus 16 terms that attempt to capture the interaction between cardiac and respiratory processes (Brooks et al., 2008; Harvey et al., 2008). It is important to note that the relative phases for each slice (e.g. position in the cardiac cycle at time of acquisition, used to calculate the physiological regressors), were calculated independently and modelled separately in the GLM. Only one out of 57 subjects lacked physiological recordings, due to equipment failure – this subject was not excluded as it was considered unlikely to increase false-positive rate in the final sample. Local autocorrelation correction was performed using FILM (Woolrich et al., 2001) as part of model estimation, which also attempted to correct for physiologically driven signals (originating from cardiac/respiratory processes) using slice-dependent regressors in PNM (FSL). Relative mean motion was extracted from each subject to look for excessive head movement. The average motion across subjects was 0.068 mm, ranging from 0.02 to 0.27. Since no subject moved more than half a voxel (i.e. 0.75 mm), no one was excluded on this basis.

#### First level analyses

The four conditions (easy|high, hard|high, easy|low, hard|low) and tasks of no interest (cues and rating periods) were modelled using a hemodynamic response function (gamma basis function, σ = 3 s, mean lag = 6 s) alongside the physiological regressors within the general linear model in FEAT (Jenkinson et al., 2012). A separate analysis tested for an intra-subject parametric relationship between pain ratings (one per block) and BOLD signal (Büchel et al., 1998). In addition to tasks of no interest and physiological signal regressors, a constant regressor for all blocks (weighting = 1) and a regressor weighting the individual pain ratings for each block were included. None of the regressors were orthogonalised with respect to any other.

#### Second level analyses

Main effects were specified as positive and negative main effect of attention (hard versus easy task, and vice versa) and positive and negative main effect of temperature (high versus low thermal stimulus, and vice versa). A task x temperature interaction contrast was also specified. The parametric data was assessed using a simple group average – to examine whether the linear relationship between pain ratings and brain activity was consistent across the group. Lastly, a paired analysis compared activity during the easy|high and hard|high conditions - to examine whether the inter-subject difference in average pain ratings (i.e. easy|high minus hard|high) was linearly related to the corresponding difference in BOLD signal (similar to Tracey et al., 2002 and Brooks et al., 2017). To test for differences between the discovery cohort and the validation cohort, we used an unpaired t-test with FLAME (height threshold *z* > 3.09, corrected cluster extent threshold *p* < 0.05), in line with guidelines on corrections for familywise error (FWE) (Eklund et al., 2016). Subsequent analyses of the pooled cohort (i.e. all 57 subjects) used the same threshold.

#### Brainstem-specific analyses

Detecting activation in the brainstem is non-trivial due to its susceptibility to physiological noise and artefacts (Brooks et al., 2013), small size of structures of interest and relative distance from signal detectors in the head coil. Consequently, a brainstem focussed analysis was performed at the group level using a series of anatomical masks and statistical inference using permutation testing (Nichols and Holmes, 2002) in RANDOMISE (part of FSL). Analyses utilised pre-defined regions of interest based on (i) a whole brainstem mask derived from the probabilistic Harvard-Oxford subcortical structural atlas (Desikan et al., 2006) and thresholded at 50% and (ii) previously defined probabilistic masks of the a priori specified brainstem nuclei (RVM, LC, PAG) from Brooks et al. (2017). The number of permutations were set to 10,000 in line with guidelines (Eklund et al., 2016) and results reported using threshold free cluster enhancement (TFCE) corrected *p* < 0.05 (Smith and Nichols, 2009).

#### Psycho-physiological interactions (PPI)

Effective connectivity analyses were performed on the pooled cohort. We used generalised PPI (gPPI) to detect changes in interactions between regions during specific experimental conditions (O'Reilly et al., 2012; McLaren et al., 2012; Friston et al., 1997). In this technique a physiological signal (e.g. the time-course extracted from a seed region) is convolved with a modelled psychological variable (i.e. each one of the experimental conditions) to build interaction regressors. All interaction regressors were added to a general linear model (GLM) that also included the non-convolved experimental conditions and tasks of no interest (e.g. the rating period). Contrasts were built to test for connectivity differences that could be explained by the main effects of task and temperature and the task * temperature interaction.

Four regions identified by the main effect analyses (temperature and/or attention) and inter-subject analgesic regression model in the pooled cohort, were selected as seed-regions for the gPPI analysis: PAG, right LC and ACC in the main effect of task and RVM in the main effect of temperature. For each subject, the physiological BOLD time course was extracted from the peak voxel of the pre-processed images (as described in the section ‘Image Pre-processing’) within each functional mask, and gPPI performed at the first level. Subsequently, group responses were estimated with permutation testing within the same functional masks e.g. effective connectivity between PAG seed region and the other three regions (RVM, right LC, ACC). To aid interpretation of significant results in the task * temperature interaction contrast, we focussed on the conditions of interest (i.e. easy|high and hard|high). Parameter estimates were extracted by first defining a sphere of radius 2 mm at the voxel of greatest significance in the group gPPI result, then back-transforming this mask to subject space and extracting the signal from the voxel with highest Z-score.

In summary, the procedure for gPPI analysis was:

• Pre-processing of functional data
• Time series extraction from functional masks
• Convolution of time-series with experimental condition
• Contrasts of interest tested using GLM via first level (single subject) analysis
• Group analysis permutation testing with functional masks
• Extraction of parameter estimates from the conditions of interest.

#### Dynamic causal modelling (DCM)

Given the inability of gPPI to resolve the directionality of connections, we sought to extend our findings by using DCM (Friston et al., 2003). This technique allows the specification of a hypothetical network model (based on Eq. (1)) fitted to the fMRI data to resolve connection strengths.

The change in activity of each region in a model with j inputs and n brain regions is formalized as follows:

(1) $$\frac{dx}{dt}=\left(aA+\sum_{j=1}^{n}u_{j}bB^{j}\right)x+cCu+\;\omega$$

Where:

x - neuronal state of a region (i.e. BOLD signal convolved with haemodynamic response function)
A - binary vector that defines the connectivity of x is to each of the other regions in the model,
a - vector of parameters that define the strengths of such connections,
u - external input to the model,
B - binary vector that defines whether model connections are modulated by external input,
b - vector of parameters that defines the strength of such modulation,
C - binary vector that defines whether x directly receives the external input,
c - contains parameters that regulate the strength of the received input,
ω - random neuronal noise.

Note since the model is estimated in a Bayesian framework, parameters are not single values but are posterior densities.

Given the results of the PPI analysis, we specified bi-linear, one state, stochastic, input centred DCMs (Daunizeau et al., 2009, 2012) in SPM 12 (Wellcome Trust Centre for Neuroimaging, London, UK). The models were estimated on a computer cluster (BlueCrystal) in the Advanced Computing Research Centre, University of Bristol – http://www.bristol.ac.uk/acrc/. Random effects Bayesian Model Selection (BMS) was used to compare the models and Protected Exceedance Probability, the likelihood of a given model in respect to the others tested, was calculated. Bayesian Omnibus Risk, a measure of the risk of all models having the same frequency within the population, was also computed (Rigoux et al., 2014). Bayesian model averaging (Penny et al., 2010) was used to extract the parameter estimates of interest.

## Results

### Comparison of the discovery cohort and validation cohort

The behavioural and imaging datasets from the validation and discovery cohort were quantitatively compared as criteria to justify the decision to pooling the two together for subsequent analyses. A three-way repeated measures ANOVA was carried out on the pain scores using task and temperature as within subject factors and the group (discovery vs validation cohort) as between subject factor. This analysis showed no effect of group on the effects of temperature (*P* = 0.481), nor task (*P* = 0.833), nor on the task*temperature interaction (*P* = 0.481), indicating that the two groups are comparable in terms of the behavioural effect.

An unpaired *t*-test on the functional image contrasts did not show any statistically significant differences between the discovery and validation cohorts for the main effect of temperature (positive and negative), main effect of task (positive and negative) and interaction contrast (positive and negative). Given the lack of demonstrable statistical differences between the two cohorts, we went ahead with our planned intention to combine the three datasets and all subsequent results relate to the pooled cohort comprising 57 subjects. We also note that the use of strict cluster thresholds for the brain, and of permutation testing for ROI-based analyses in ‘noisy’ brainstem regions, can produce robust and reproducible results even with a sample size of 20 (Brooks et al., 2017).

### Behavioural analysis (Pooled cohort)

The average high (noxious) temperature in the pooled cohort was 43.4 °C (range 42 °C - 45 °C). Analysis of the pain ratings showed the expected main effect of temperature on pain scores (F(1, 56) = 252.799, *P* < 0.0001, repeated measures ANOVA) but no main effect of task (F(1,56) = 2.935, *P* = 0.092). There was a clear task x temperature interaction (F(1, 56) = 31.969, *P* < 0.0001, Fig. 1) and post-hoc paired *t*-test showed performance of the hard task produced a decrease in pain scores in the high temperature condition (mean hard|high = 38.1, SD 17.0 vs easy|high = 42.1, SD 16.5, *P* < 0.0001, Bonferroni corrected), consistent with an attentional analgesic effect (Fig 1).

Additional exploratory analysis did not detect any evidence of an order effect in the pain ratings (F(3165) = 0.164, *P* = 0.92 one-way repeated measures ANOVA), meaning that we did not observe a significant sensitisation or habituation in subjects’ pain ratings. Similarly, there was no effect of gender on attentional analgesia (F(1,55)=0.091, *P* = 0.764), nor on the main effects of temperature (F(1,55)=1.69, *P* = 0.198) or task on pain ratings (F(1,55)=0.253, *P* = 0.617, all mixed model ANOVAs).

### Whole brain & brainstem-focussed analysis (Pooled cohort)

**Fig. 2:**
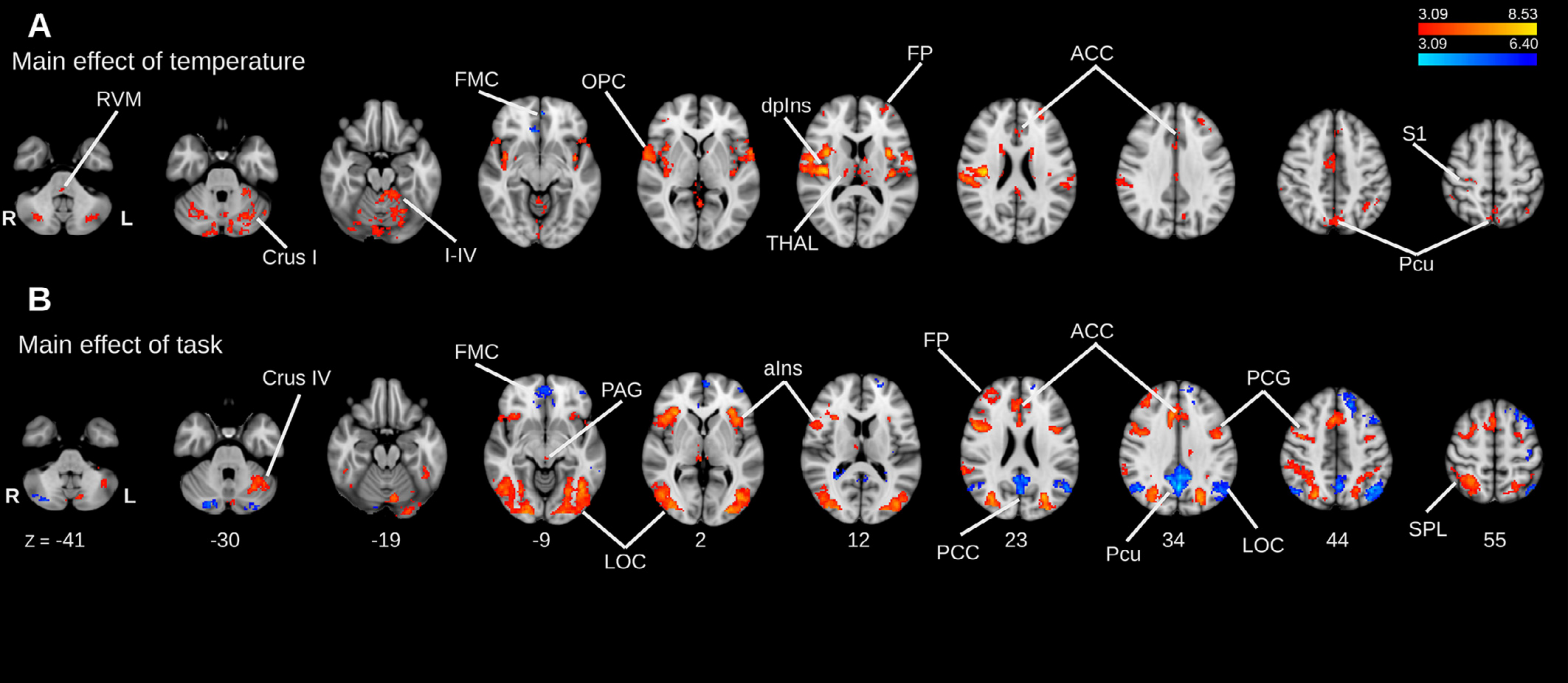
*Whole brain main effect analyses in the pooled cohort (N* *=* *57). Positive (red/yellow) and negative (blue/light-blue). Data was obtained from cluster-based thresholding using an initial threshold of Z > 3.09 and FWE corrected p < 0.05, one-sample t-test.****(A)****Main effect of****temperature****. Positive activation in the high temperature conditions was found in anterior cingulate cortex (ACC), thalamus (THAL), dorsal posterior insula (dpIns), precuneus (Pcu), primary somatosensory cortex (S1) and rostroventromedial medulla (RVM). Activation in the negative main effect of temperature (low temperature* vs *high temperature) was observed in the frontal medial cortex (FMC).****(B)****Main effect of****task****. Activity in the positive main effect was found in the anterior insula (aIns), lateral occipital cortex (LOC), ACC, superior parietal lobule (SPL). Activity in the negative main effect was found in the frontal pole (FP), posterior cingulate cortex (PCC) and Pcu.*

**Fig. 3:**
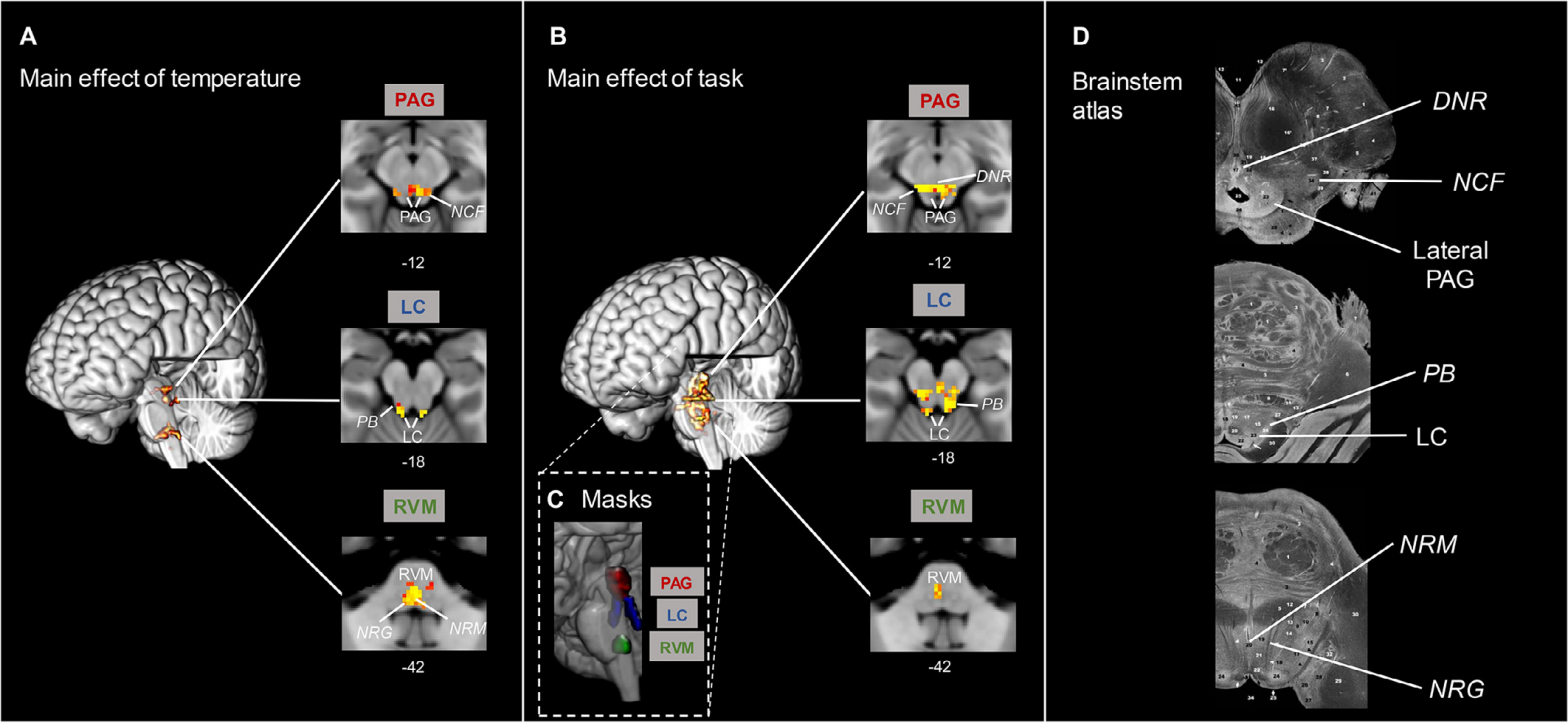
*Main effect analyses in the brainstem. Results obtained after permutation testing with a probabilistic whole brainstem mask (p < 0.05, TFCE corrected).****(A)****Clusters of activation in the brainstem corresponding to the main effect of****temperature****included the ventral and lateral periaqueductal grey (PAG), rostral ventromedial medulla (RVM), including nucleus raphe magnus (NRM) and nucleus reticularis gigantocellularis (NRG), bilateral locus coeruleus (LC) which were all localised using the masks in (C) and activation was also noted in the region of the dorsal nucleus of raphe (DNR), nucleus reticularis cuneiformis (NCF) and parabrachial nucleus (PB) identified by comparison with the Duvernoy brainstem atlas (**Naidich et al., 2009**).****(B)****Extensive brainstem activity was observed in the main effect of****task****, including the PAG, RVM (to a lesser extent than for****temperature****), bilateral LC (all localised using regional masks), as well as activity in the region of DNR, NCF and PB.****(C)****Shows the position and extent of the anatomical masks defined in (Brooks* et al.*, 2017) used to confirm the localisation of activity in the PAG (red), LC (blue), RVM (green).****(D)****shows comparative MR microscopy sections from the Duvernoy brainstem atlas (reproduced with permission), modified to highlight activity identified by reference to the atlas (shown in italics).*

Activations were found for the positive main effect of temperature in a range of regions including the anterior and posterior cingulate cortices, precuneus, cerebellum, post-central gyrus (S1), dorsal posterior insula and opercular cortex, in the latter three cases with more prominent clusters contralateral to the side of thermal stimulation (Fig. 2A). In the negative main effect of temperature, significant clusters were found in the frontal medial cortex and in the subcallosal cortex (Fig 2A). We also found a cluster of activation in the RVM at this whole brain level. However, to improve our ability to resolve activity in hindbrain structures, we undertook permutation testing using a whole brainstem mask, which revealed, among the others, clusters of activation in the positive main effect of temperature in the ventral PAG, LC bilaterally as well as the RVM (Fig 3A, *p* < 0.05, TFCE corrected), identified with anatomical masks previously defined (Brooks et al., 2017, Fig 3C). These brainstem clusters spanned beyond our anatomical masks, with activity originating from other brainstem nuclei, such as nucleus cuneiformis and parabrachial nucleus. The latter nuclei were visually identified with the aid of the Duvernoy's Atlas (Fig 3D, Naidich et al., 2009).

Analysis of the positive main effect of task, showed extensive areas of activation within the lateral occipital cortex, superior parietal lobule, anterior cingulate cortex and anterior insula, as well as the PAG (Fig 2B). In the negative main effect of task, clusters were located in the posterior cingulate cortex, frontal medial cortex and in the lateral occipital cortex (Fig 2B). Permutation tests within the whole brainstem masque showed multiple clusters of activation, including in the LC bilaterally, RVM and PAG, identified with anatomical masks (Fig 3B-C, *p* < 0.05, TFCE corrected). In addition, significant brainstem activity was identified in other brainstem nuclei, including dorsal nucleus of raphe, nucleus cuneiformis, and parabrachial nucleus, identified with the Duvernoy's Atlas (Fig 3D, Naidich et al., 2009)).

In the interaction contrast between task and temperature no cluster reached significance either at the whole brain level nor when using the whole brainstem masked analysis.

These findings from the pooled cohort showed close similarity to those of Brooks et al. (2017) with the same areas found in the main effects analysis (Supplementary Table 1 shows all significant clusters). The additional findings at a whole brain level were that both the RVM and the precuneus now appear in the main effect of temperature and the dorsolateral PAG in the main effect of task (the RVM and PAG were only seen in a nucleus specific masked analysis in Brooks et al., 2017). Similarly, activity in the brainstem is now seen in more areas using a whole brainstem mask rather than only in the nucleus specific masks (e.g. main effect of temperature in RVM alone previously versus RVM, LC and PAG in this pooled analysis).

Whilst the patterns of activity within the cerebrum were largely non-overlapping, there were some areas which appeared to be common to both the main effect of task and temperature: ACC, FMC and cerebellum. To formally test the degree of overlap, we performed a conjunction analysis (Friston et al., 1999) which revealed that of the hypothesised brain regions involved in the task, only the ACC was active in both conditions (cluster forming threshold *Z* > 3.09, FWE corrected *p* < 0.05).

### Linear encoding of pain intensity

**Fig. 4:**
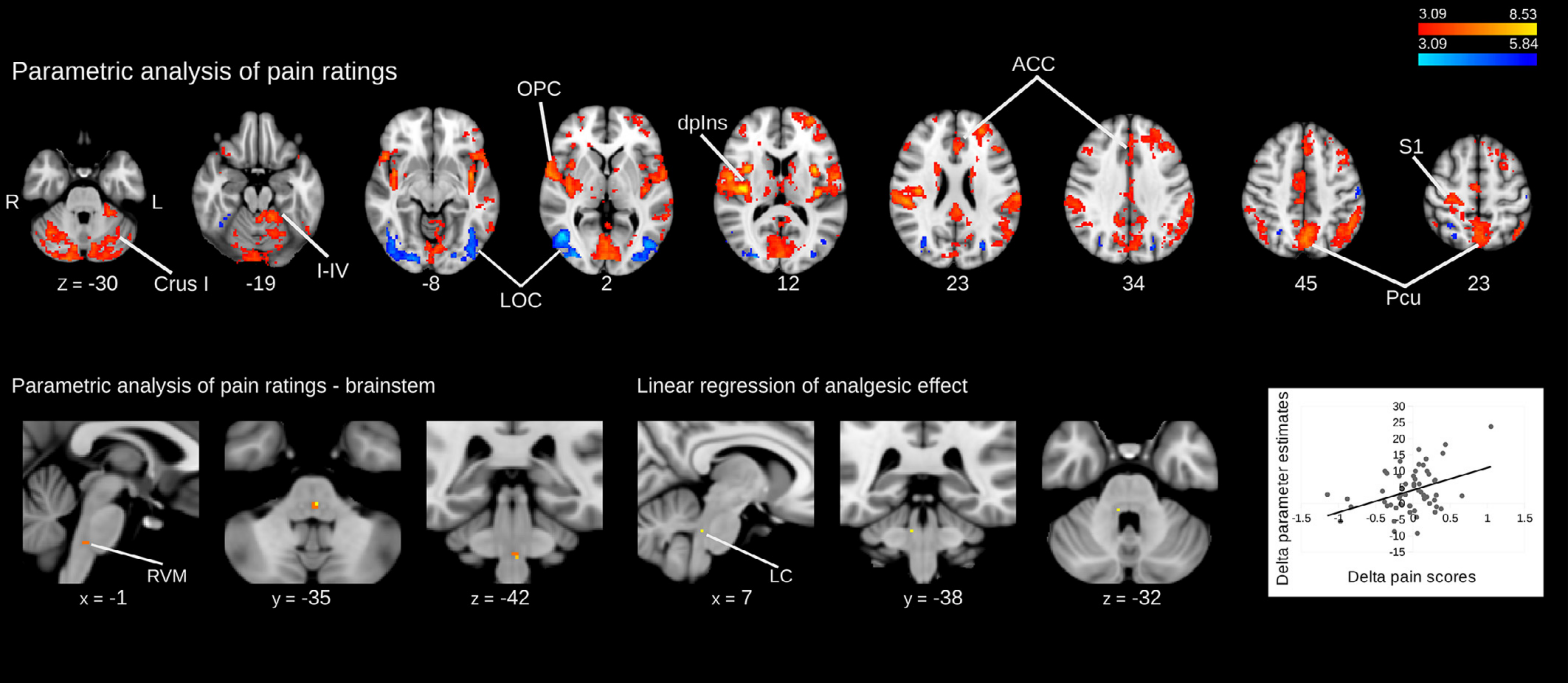
***A)****Pain encoding regions were identified by intra-subject parametric regression with pain ratings across all the experimental conditions, in the whole brain analysis. Regions whose activity linearly increased with perceived pain are shown red-yellow and regions whose activity decreased with perceived pain in blue-light blue. (height threshold Z > 3.09, corrected cluster extent threshold p < 0.05)****B)****Brainstem intra-subject parametric regression with pain ratings, using RVM, LC and PAG masks. Only the RVM showed a linear increase in activity with the pain scores (p < 0.05, TFCE corrected).****C)****Brainstem inter-subject regression with magnitude of analgesia (*i.e. *difference in pain ratings for the two high temperature conditions easy|high minus hard|high****).****The right (contralateral) LC was the only region whose activity correlated with the difference in pain ratings. Data obtained using an LC mask (p < 0.05, TFCE corrected).*

Brain regions whose activity was linearly related to perceived pain intensity were identified using an intra-subject parametric regression. This revealed a network of positively correlated regions (similar to those seen in the main effect of temperature) including primarily the right (contralateral) dorsal posterior insula and S1, the anterior cingulate cortex, frontal lobe and the precuneus (Fig 4A). Regions showing a linear decrease in activation with pain ratings were restricted to the occipital cortex bilaterally and ipsilateral primary somatosensory cortex (Fig 4A). Permutation testing in the brainstem (using RVM, PAG and LC masks) identified only the RVM as showing a positive correlation with pain intensity (Fig 4B). No brainstem region showed a negative correlation with pain. All these findings were consistent with Brooks et al. (2017), with the addition of a cluster identified in the thalamus (Supplementary Table 2).

### Regions whose activity correlates with analgesic effect

An inter-subject whole-brain mixed effects comparison between the hard|high and easy|high conditions did not identify any region whose activity linearly correlated with the differences in pain ratings (i.e. analgesia). A parametric regression showed a linear relationship between difference in activity and analgesic effect in only the contralateral (right) LC (i.e. decreased pain ratings were associated with increased BOLD difference), after permutation testing with LC, RVM and PAG masks (Fig. 4C). A positive relationship was noted between the difference (“Delta”) in parameter estimates extracted from the rLC and the attentional analgesic effect on pain scores (Fig 4C).

### gPPI analysis between neural hubs linked to attentional analgesia

**Fig. 5:**
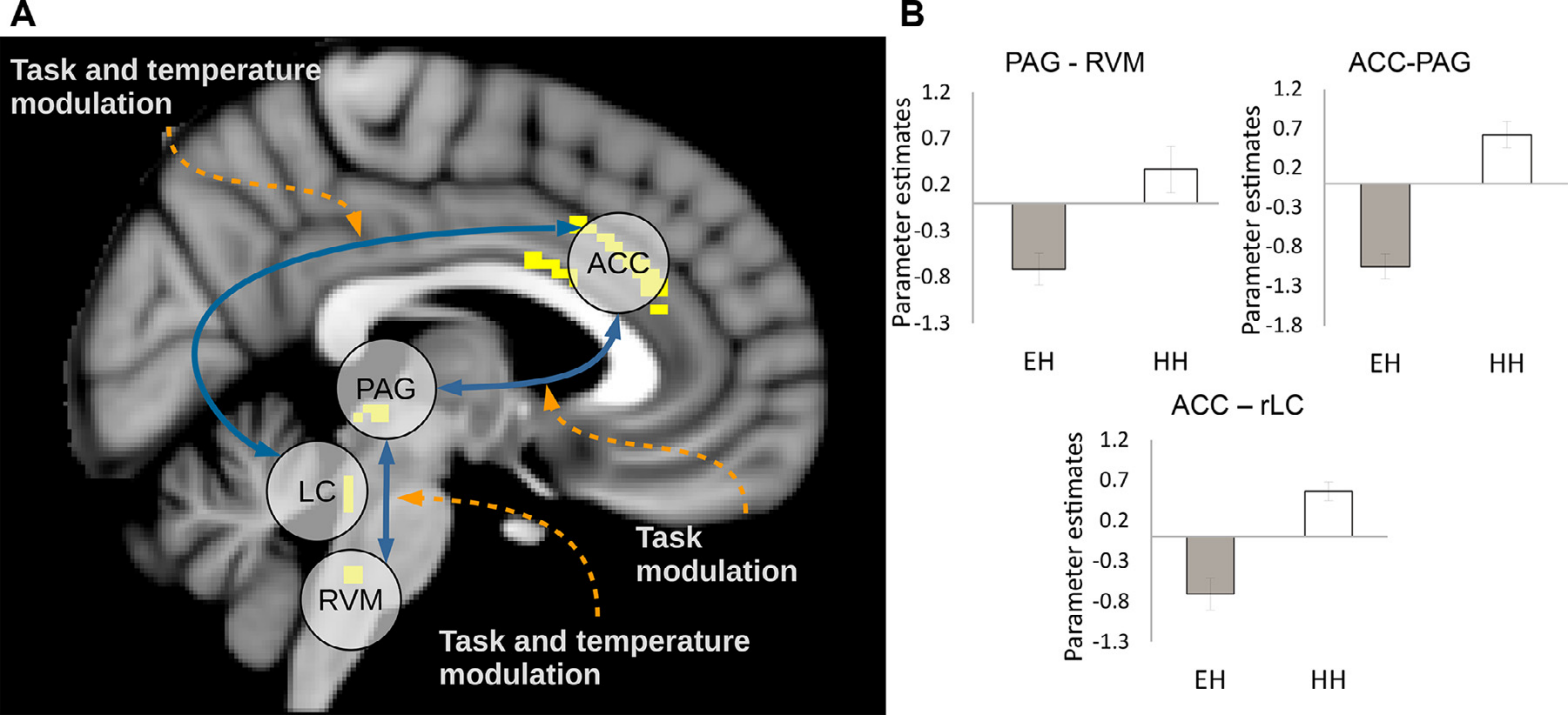
***(A)****Schematic representation of results of the gPPI analysis. Results were obtained with single-region functional masks and permutation testing (P < 0.05, TFCE§ corrected).****(B)****Parameter estimates extracted from the peak destination voxel from the PPI analysis (see text for details), in the easy|high and hard|high conditions. Note that all arrows are double-headed as it is not possible to determine the directionality of connections with gPPI analysis.*

To determine the changes in neuronal communication associated with attentional analgesia, this analysis aimed to identify changes in effective connectivity associated with task difficulty, temperature and the task x temperature interaction. Results from main effects, conjunction and parametric analgesia analyses provided the motivation for selecting a subset of the activated brain regions, that were subsequently used for connectivity analysis. Time courses were extracted from functional masks for gPPI analyses: RVM for the main effect of temperature, and PAG, rLC and ACC for the main effect of task (see Methods). Permutation testing revealed increased connectivity with the following contrasts (see Fig 5):

• RVM seed - increased connectivity to PAG for the interaction contrast
• ACC seed - increased connectivity with the right (contralateral) LC in the interaction contrast and with the PAG in the main effect of task
• PAG seed - did not show any significant change in effective connectivity
• rLC seed - did not show any significant change in effective connectivity.

For all gPPI results, parameter estimates were extracted from the voxel with greatest significance in each individual to explore the nature of these interactions (Fig 5B). In all cases, the parameter estimates were greater in the hard|high compared to the easy|high condition, indicating an increase in coupling in the condition associated with attentional analgesia i.e. hard|high.

### DCM to determine directionality of pathway interactions

**Fig. 6:**
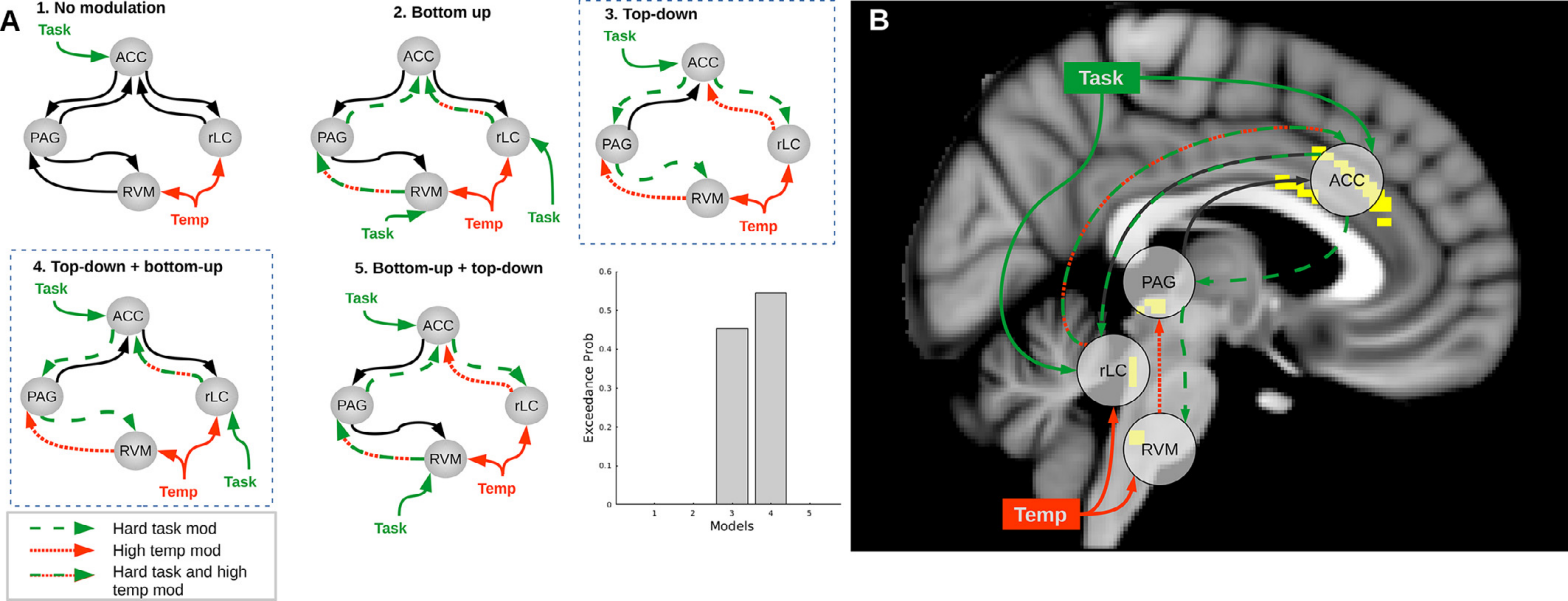
(A) Specified network interactions assessed with Dynamic Causal Modelling and result of Bayesian Model Selection. The effect of temperature is always bottom-up, while the task could have a bottom-up or top-down effect. The inputs are both easy/hard task and low/high temperature, while the modulations of connections are only high temperature and hard task. Model 3 and 4 (outlined with dashed box) have the strongest evidence of reproducing the data, with slightly stronger evidence for model 4. (B) Schematic representation of interactions during attentional analgesia, after PPI and DCM.

Dynamic causal modelling was used to resolve the directionality of the task effect on the connections identified in the gPPI. We systematically varied the location of the task inputs and modulation, while the temperature modulation was kept fixed in all models as a bottom-up effect. External inputs were both hard/easy task and high/low temperature, while modulations were only hard task and high temperature. Five models were specified (Fig 6A):

(1) No modulation of connections,
(2) Task bottom-up on the ACC-PAG-RVM and on the ACC-LC axis.
(3) Task top-down for both pathways.
(4) Task top-down in the ACC-PAG-RVM axis and bottom-up in the ACC-LC connection.
(5) Task bottom-up in ACC-PAG-RVM and top-down in ACC-LC.

Models 3 and 4 were found to best fit the data in BMS, with protected exceedance probability = 0.45 and 0.55 respectively (Fig 6B), and Bayesian omnibus risk of zero. In both, the task had a top-down influence on ACC-PAG-RVM, while the ACC-LC connection was top-down modulated in one model and bottom-up modulated in the other. Bayesian model averaging was used to extract parameter estimates (Supplementary Table 3).

All connections were also tested with an analgesic covariate, to find whether one or more consistently differed in participants that showed an analgesic effect. No connection reached significance in this test.

## Discussion

The brainstem involvement in attentional analgesia has been investigated in previous studies, demonstrating a mediating role of the PAG and of its interaction with cortical regions (Brooks et al., 2017; Tracey et al., 2002; Valet et al., 2004). However, possibly because of a lack of statistical power or technical limitations, the neuronal interactions between cortex, PAG and the lower brainstem nuclei in this context have never been fully resolved. Reassuringly, in the context of the reproducibility crisis that is afflicting neuroscience, especially in fMRI experiments (Button et al., 2013; Eklund et al., 2016), we have recapitulated the core findings regarding the brainstem hubs involved in attentional analgesia (Brooks et al., 2017) and have extended our analysis to determine how they interact to produce attentional analgesia by functional network analysis. This identifies a top-down pathway from the PAG to the RVM, engaged by cortical input from the ACC during high cognitive demand. In addition, there is a parallel bidirectional communication between ACC and LC during attentional analgesia.

### Identification of brainstem nuclei involved in attentional analgesia

The higher statistical power provided by 57 subjects, some 3-fold greater than in Brooks et al. (2017), yielded stronger findings especially in the brainstem nuclei. In the main effect of temperature, the specificity of the pattern of nociceptive information flow is striking with activations confined to discrete territories including ventral PAG, LC and RVM as well as activations in the region of parabrachial nucleus, nucleus solitarius, sub nucleus reticularis dorsalis and nucleus cuneiformis. This expands our previous result showing only RVM response to high temperature stimulation (Brooks et al., 2017). While it has long been known from animal studies that these brainstem regions receive nociceptive input from the spinal cord (Blomqvist and Craig, 1991; Cedarbaum and Aghajanian, 1978; Keay et al., 1997) this has seldom been clearly demonstrated in human imaging studies. In addition, an intra-subject linear regression analysis with pain scores revealed that the BOLD signal in the RVM linearly scales with perceived pain, in agreement with recent studies (Brooks et al., 2017; Horing et al., 2019). This clearly demonstrates that this brainstem territory is likely to be playing an important role in coding nociceptive intensity. It is possible that the voxels resolved in this analysis are related to the activity of the pro-nociceptive ON-cells in the RVM. To our knowledge, no other single study has been able to produce such a complete activation map in the human brainstem in response to noxious stimulation (see review by Henderson and Keay, 2018).

In the main effect of task, we again detected activity in PAG, RVM and LC bilaterally, in addition to a more diffuse activation in the brainstem. It is interesting to note that the attentional task recruited the dorsal and ventral PAG whereas the noxious input produced activation in the ventral region of the nucleus, perhaps in line with the known behavioural specialisation of columns within this crucial integrating nucleus (Linnman et al., 2012; Roy et al., 2014).

The magnitude of the analgesic effect showed a correlation with activity in the right LC (a finding that we previously noted in Brooks et al. (2017) but was just below formal statistical significance). This was the only location in the neuroaxis that showed this relationship, and is the reason why connectivity analysis focused on the right LC. One intriguing aspect of this interaction is the lateralised nature of the relationship between the right LC (i.e. contralateral to the stimulus) and the analgesic effect - a finding that has previously been noted in rodent studies where noxious stimuli increase the activity in the contralateral LC to a greater effect (Cedarbaum and Aghajanian, 1978). The LC is well positioned both anatomically and functionally to mediate a component of attentional analgesia, not only because it is responsive to attentional states and cognitive task performance (Aston-Jones and Cohen, 2005; Sales et al., 2019; Sara, 2009; Yu and Dayan, 2005) and to nociceptive inputs (Cedarbaum and Aghajanian, 1978; Howorth et al., 2009), but also particularly in being able to cause analgesia via its direct spinal cord projections (Hirschberg et al., 2017; Jones and Gebhart, 1986). Intriguingly, the spinal cord-projecting neurons are located in the caudal part of the LC in rodents (Hirschberg et al., 2017), as is the LC region that we found to correlate with the analgesic effect. Previous studies have demonstrated linear relationships between the analgesic effect and activity located in the PAG (Tracey et al., 2002, in 9 subjects) and RVM (Brooks et al., 2017, in 20 subjects). We note that neither of these findings were replicated in our current study of 57 subjects. While all three of these regions have biological plausibility for mediating analgesia, a larger sample size seems necessary to produce robust results with inter-subject regression (especially in small, noisy brainstem nuclei) and this is likely to be complicated by the known interactions between these regions in nociceptive processing (discussed below).

### Parallel cortical-brainstem pathways

Given the involvement of PAG, RVM and LC in both aspects of the experiment, and their known involvement in endogenous analgesia (Ossipov et al., 2010), we tested all three nuclei for connectivity changes during the attentional analgesia paradigm.

Cortical regions involved in the endogenous modulation of pain in humans include the anterior cingulate cortex, the dorsolateral prefrontal cortex and the ventrolateral prefrontal cortex (Bushnell et al., 2013). Among these, the ACC was the only frontal cortical area showing activity in the conjunction analysis between main effects of task and temperature, and prior evidence showed its interaction with the PAG to be involved in attentional analgesia (Valet et al., 2004). In light of the recent discussions around compartmentalisation of the cingulate (Van Heukelum et al., 2020), it should be acknowledged within this framework that our results pertain to both MCC (involved in conflict resolution between competing attentional demands) and ACC (nociceptive, affective processing). The location of the ACC region resolved here is indeed on the ACC-MCC border, where activity likely reflects a combination of task demand and pain processing. Intriguingly, inputting the coordinates of the peak attentional activation of the ACC to Neurosynth (Yarkoni et al., 2011) identified four studies where the same region was involved in response to conflict (Barch et al., 2001; Scholl et al., 2017; van Veen and Carter, 2005; Wittfoth et al., 2008). In addition, voluntary control over the activation of this area was shown to result in modulation of pain perception in a neurofeedback study (deCharms et al., 2005).

To examine the interplay between the cortical and brainstem structures we hypothesised to be involved in attentional analgesia, we initially performed a generalised PPI, which determines how connectivity changes as a result of experimental manipulation (i.e. effective connectivity). We observed altered connectivity between the ACC and contralateral (right) LC during the interaction between task and temperature. Furthermore, coupling increased between ACC and PAG with task demand, and between PAG and RVM during the task x temperature interaction. Extraction of parameter estimates revealed that all interactions were enhanced in the hard task/high temperature condition.

The identified network interactions lacked directionality and could equally be evidence for an ascending pathway, where the attentional demand modulates how the nociceptive information reaches the brain, or a descending pathway, where the cortex recruits brainstem nuclei to modulate the spinal cord. Therefore, dynamic causal modelling was employed to explore these hypotheses by fitting different models to the data. Bayesian Model Selection validated the results of the gPPI by excluding, for lack of evidence, a model where no connection was modulated by task. In addition, BMS resolved a top-down influence of task on the ACC-PAG and PAG-RVM connections, consistent with a descending pain modulatory system involved in attentional analgesia (Sprenger et al., 2012). The ACC-LC pathway was however not resolved as clearly, with similar evidence in BMS for task modulation of the top-down and bottom-up connection. On examination of the parameter estimates, it was noted that the task modulation had a negative effect on all connections that were also modulated by temperature. Conversely, the ACC-PAG connection, only modulated by task, has a positive parameter estimate. This effect suggests a disinhibitory effect, or a negative feedback loop in the PAG-RVM and ACC-LC connections. Neurobiological mechanisms that could account for these effects are discussed below.

Effective connectivity changes in these pathways may mediate the process of attentional analgesia. This could be achieved through LC projections to the ACC increasing the signal-to-noise (or salience) of one input over another (Manella et al., 2017; Muller et al., 2019; Sales et al., 2019; Sara, 1985; Vazey et al., 2018) and/or ACC to spinally projecting LC neurons modulating the activity of dorsal horn neurons (i.e. decreasing nociceptive transmission), both actions potentially giving 'precedence' to the task. It is possible that the ACC and the LC work in a reciprocal negative feedback loop during attentional analgesia (Breton-Provencher and Sur, 2019; Ramos and Arnsten, 2007). The reduction in perceived pain could equally be achieved via ACC recruiting the PAG and RVM to produce antinociception at a spinal level during the attention demanding task (Millan, 2002), for example by disinhibition of the RVM “off-cells” (Lau and Vaughan, 2014). This conceptually extends previous studies that have identified the ACC-PAG connection as being involved in a distraction from pain (attentional analgesia) paradigm (Valet et al., 2004), as well as in a placebo analgesia paradigm (Petrovic, 2002). The PAG-RVM descending control system has also already been implicated in placebo analgesia (Eippert et al., 2009; Grahl et al., 2018) via an opioid-dependent mechanism. The behavioural component of attentional analgesia has been reported to be impaired by opioid blockade, possibly by disrupting connections between the ACC-PAG-RVM descending control system (Sprenger et al., 2012). It is also quite conceivable that the parallel ACC-LC and ACC-PAG-RVM systems described here work in concert to cause analgesia. Previous animal studies show that electrical stimulation of the PAG triggers noradrenaline release in the cerebrospinal fluid and the analgesic effect of stimulation can be partially blocked with intrathecal alpha2-antagonists (Cui et al., 1999; Hammond et al., 1985). In addition, it was demonstrated that mice not able to synthesize noradrenaline were less sensitive to the analgesic action of morphine (Jasmin et al., 2002). Thus, it still remains to be demonstrated whether these two pathways are working in a parallel independent fashion, or are dependent upon each other in producing attentional analgesia.

We propose that the ACC acts to resolve the conflict caused by an attention-demanding painful stimulus and the cognitive load of a sustained visual attention task, by sending downstream signals to brainstem structures to facilitate optimal behaviour. This interpretation is in accordance with previous hypotheses on the function of the ACC-LC interaction, implicated in re-orienting attentional processes (Corbetta et al., 2008). In addition, recent evidence from a human fMRI study identified the same connection during conflict resolution in an incongruent Stroop task (Köhler et al., 2016).

We propose that this network could be relevant for mindfulness-based analgesic techniques, especially the “focused attention” type, where focus on an internal signal (e.g. breathing), can distract subjects from pain (Zeidan et al., 2011). While this might be only one of the mechanisms to meditation analgesia, it is worth mentioning that this process is not mediated by endogenous opioids (Zeidan et al., 2016) but relies on the rACC (Zeidan et al., 2012; Zeidan and Vago, 2016), perhaps by exclusively engaging the ACC-LC pathway.

We further postulate that this network may be of importance in chronic pain conditions (e.g. fibromyalgia), where disruption of attention and cognition are co-morbid alongside pain. Pharmacological therapies that target the noradrenergic system have some benefit in chronic pain conditions (Hughes et al., 2015; Kremer et al., 2016, 2018), possibly by acting on the LC system (Hiroki et al., 2017). On the other hand, evidence for malfunction of endogenous pain modulation in such pathologies (Julien et al., 2005; Lannersten and Kosek, 2010; Staud et al., 2005; Vierck et al., 2001), together with the evidence of low effectiveness of opioid drugs (Goldenberg et al., 2016; Kia and Choy, 2017), might point toward impairments of the PAG-RVM interaction.

### Methodological considerations

Because of the increased sample size, we were able to detect activation in the RVM and PAG in the main effect of temperature and task respectively, without the aid of masking. This experimentally validates the results in Brooks et al. (2017) as well as the use of permutation testing with anatomical masks of a-priori specified ROIs. Notwithstanding the difficulty of accurately assigning measured functional activity to specific brainstem nuclei (Betts et al., 2019; Keren et al., 2009; Tona et al., 2017) and the problems faced when trying to image these structures (Brooks et al., 2013), the ability to corroborate our earlier findings should provide confidence for future studies of the brainstem. However, there is still a clear and pressing need for an objectively defined probabilistic brainstem atlas, as exists for other brain structures (Kurth et al., 2010).

We used gPPI analysis, a well-established technique in the neuroimaging field, for network discovery. The strength of gPPI is the ability to detect functional changes in the interaction between two regions, caused by experimental manipulation. This is different from a seed-based analysis that detects functional interactions between regions that remain constant during the whole acquisition period. We then used DCM with the singular purpose of resolving the directionality of the connections (after (Yoshino et al., 2010)), . DCM can be used on its own for network discovery, with a larger model space that tests all possible connections and modulations. However, a large model space is likely to cause a dilution of model evidence, leading to less clear results. In addition, the complexity (e.g. the number of connections) was kept constant across models, to avoid the risk of overfitting.

We employed stochastic DCM, which allows for modelling of random neuronal noise in the system, to improve network resolution in brainstem areas significantly affected by physiological noise (Brooks et al., 2013). This routine was shown to improve the characterization of network structure and parameter inference over deterministic DCM (Daunizeau et al., 2012; Osório et al., 2015) and has been widely used in resting state and task-based fMRI studies since its release (Kahan et al., 2014; Ma et al., 2015, 2014; Ray et al., 2016; Zhang et al., 2015).

## Conclusion

In this study we have been able to resolve parallel cortical – brainstem pathways that form a network that is functionally engaged when pain perception is attenuated during attentional analgesia. We note that the spinal cord BOLD response to nociception has previously been shown to be modulated by attention (Sprenger et al., 2012). Whether this spinal modulation of nociception is the product of activation of the ACC-PAG-RVM and/or the ACC-LC system still needs to be demonstrated in humans. It is known that both pathways could involve opioids (Fields, 2004) and so previous studies using naloxone do not discriminate between these possibilities. It would be interesting to explore whether conflict resolution resulting in attentional analgesia is dependent on the ACC-LC interaction or it could be achieved independently via the ACC-PAG-RVM path. A connectivity analysis examining the network activity between cortical territories, brainstem nuclei and dorsal horn in toto may help to define the key pathway in attentional analgesia.

## CRediT authorship contribution statement

**Valeria Oliva:** Conceptualization, Resources, Data curation, Software, Formal analysis, Validation, Investigation, Visualization, Methodology, Writing - original draft, Writing - review & editing. **Rob Gregory:** Conceptualization, Data curation, Formal analysis, Validation, Investigation, Visualization, Methodology. **Wendy-Elizabeth Davies:** Data curation, Formal analysis, Validation, Investigation, Visualization, Methodology, Project administration. **Lee Harrison:** Data curation, Formal analysis, Validation, Investigation, Visualization, Methodology. **Rosalyn Moran:** Conceptualization, Resources, Formal analysis, Supervision, Funding acquisition, Investigation, Methodology, Writing - original draft, Writing - review & editing. **Anthony E. Pickering:** Conceptualization, Resources, Formal analysis, Supervision, Funding acquisition, Investigation, Writing - original draft, Project administration, Writing - review & editing. **Jonathan C.W. Brooks:** Conceptualization, Resources, Formal analysis, Supervision, Funding acquisition, Investigation, Methodology, Writing - original draft, Project administration, Writing - review & editing.

## Appendix Supplementary materials

Image, application 1

Image, application 2
