# Supplementary material for: Parallel cortical-brainstem pathways to attentional analgesia
Source: Neuroimage. 2021 Feb 1;226:117548. doi: 10.1016/j.neuroimage.2020.117548 (PMC7836236; doi:10.1016/j.neuroimage.2020.117548)
Supplement: Supplementary file 1 [file mmc1.docx]

| Voxels | Z-MAX | X (mm) | Y (mm) | Z (mm) | Atlas labels |
| --- | --- | --- | --- | --- | --- |
| **Main effect of temperature in the pooled cohort** | | | | | |
| 2680 | 8.53 | 40 | -18 | 18 | 46% Central Opercular Cortex, 27% Parietal Operculum Cortex, 5% Insular Cortex |
| 2072 | 5.08 | 6 | -86 | -24 | 3% Occipital Fusiform Gyrus |
| 839 | 7.03 | -36 | 4 | 12 | 61% Central Opercular Cortex, 10% Insular Cortex |
| 386 | 4.87 | 0 | -70 | 48 | 76% Precuneous Cortex |
| 352 | 4.84 | 0 | 20 | 28 | 87% Cingulate Gyrus, anterior division |
| 345 | 6.58 | -38 | -18 | 18 | 50% Central Opercular Cortex, 20% Insular Cortex, 5% Parietal Operculum Cortex |
| 268 | 5.26 | 22 | -46 | 72 | 54% Superior Parietal Lobule, 12% Postcentral Gyrus |
| 265 | 5.23 | 32 | -26 | 62 | 35% Precentral Gyrus, 28% Postcentral Gyrus |
| 238 | 4.75 | 4 | -26 | 8 | 28.2% Right Thalamus |
| 223 | 4.87 | -28 | 54 | 18 | 85% Frontal Pole |
| 133 | 4.31 | 4 | -26 | 30 | 81% Cingulate Gyrus, posterior division |
| 97 | 4.09 | 12 | -10 | 18 | 55.0% Right Thalamus |
| 95 | 4.7 | -34 | -56 | 42 | 29% Superior Parietal Lobule, 18% Angular Gyrus, 13% Supramarginal Gyrus, posterior division, 12% Lateral Occipital Cortex, superior division |
| 43 | 4.25 | 20 | 16 | 18 | 5.8% Right Caudate |
| 42 | 4.47 | 36 | 46 | 8 | 47% Frontal Pole |
| 41 | 4.34 | 2 | -90 | -6 | 34% Lingual Gyrus, 23% Occipital Pole, 13% Intracalcarine Cortex |
| 39 | 4.73 | -26 | -50 | -50 | 57.0% Left VIIIa, 35.0% Left VIIIb |
| 36 | 3.97 | -34 | 32 | 40 | 66% Middle Frontal Gyrus, 7% Frontal Pole |
| 34 | 4.54 | -16 | 8 | 22 | 25.6% Left Caudate |
| 34 | 4.11 | -48 | -54 | 46 | 44% Angular Gyrus, 25% Supramarginal Gyrus, posterior division, 9% Lateral Occipital Cortex, superior division |
| 32 | 4.8 | 36 | -82 | -22 | 7% Lateral Occipital Cortex, inferior division |
| 32 | 4.33 | 48 | -44 | 50 | 46% Supramarginal Gyrus, posterior division, 17% Angular Gyrus |
| 29 | 4.31 | -4 | -40 | -44 | 100.0% Brain-Stem |
| 26 | 4.3 | -48 | -64 | -30 | 99.0% Left Crus I |
| **Negative main effect of temperature in the pooled cohort** | | | | | |
| 67 | 4.25 | 6 | 30 | -6 | 21% Subcallosal Cortex, 13% Cingulate Gyrus, anterior division |
| 28 | 4.06 | -4 | 46 | -12 | 63% Frontal Medial Cortex, 28% Paracingulate Gyrus |
| **Main effect of task in the pooled cohort** | | | | | |
| 4948 | 7.22 | 22 | -90 | -8 | 29.00% Occipital Fusiform Gyrus, 22.00% Occipital Pole, 10.00% Lateral Occipital Cortex, inferior division |
| 4790 | 7.33 | -28 | -76 | 20 | 41.00% Lateral Occipital Cortex, superior division |
| 3773 | 7.13 | 46 | 16 | 0 | 48.00% Frontal Operculum Cortex, 6.00% Insular Cortex |
| 635 | 6.81 | -34 | 26 | 0 | 38.00% Frontal Orbital Cortex, 20.00% Frontal Operculum Cortex, 13.00% Insular Cortex |
| 540 | 5.26 | 36 | 38 | 36 | 56.00% Frontal Pole, 23.00% Middle Frontal Gyrus |
| 433 | 5.87 | -42 | 6 | 26 | 27.00% Precentral Gyrus, 26.00% Inferior Frontal Gyrus, pars opercularis |
| 241 | 6.33 | -8 | -74 | -38 | 43.0% Left VIIb, 38.0% Left Crus II |
| 205 | 5.43 | 4 | -26 | -2 | 12.8% Brain-Stem |
| 58 | 4.68 | -46 | -38 | 38 | 25.00% Supramarginal Gyrus, anterior division, 10.00% Supramarginal Gyrus, posterior division |
| 46 | 4.65 | 44 | -8 | -10 | 66.00% Planum Polare |
| 45 | 4.14 | -28 | -2 | 58 | 28.00% Middle Frontal Gyrus, 16.00% Superior Frontal Gyrus, 6.00% Precentral Gyrus |
| 41 | 5 | -38 | -52 | -44 | 63.0% Left Crus II, 13.0% Left VIIb, 6.0% Left Crus I |
| 38 | 5.71 | 10 | -74 | -38 | 57.0% Right Crus II, 18.0% Right VIIb |
| 27 | 4.52 | -14 | -22 | 36 | 19.00% Cingulate Gyrus, posterior division |
| **Negative main effect of task in the pooled cohort** | | | | | |
| 1731 | 6.31 | 8 | -56 | 30 | 38% Precuneous Cortex, 20% Cingulate Gyrus, posterior division |
| 1126 | 6.6 | -38 | -72 | 48 | 68% Lateral Occipital Cortex, superior division |
| 876 | 5.43 | -34 | 18 | 56 | 63% Middle Frontal Gyrus, 2% Superior Frontal Gyrus |
| 511 | 5.77 | 32 | -70 | -36 | 44.0% Right Crus I, 24.0% Right Crus II |
| 461 | 5.95 | 50 | -66 | 40 | 83% Lateral Occipital Cortex, superior division |
| 346 | 4.66 | 6 | 28 | -4 | 14% Subcallosal Cortex anterior division |
| 257 | 5.06 | -36 | -24 | 70 | 32% Postcentral Gyrus, 24% Precentral Gyrus |
| 125 | 4.97 | -38 | -72 | -36 | 97.0% Left Crus I |
| 94 | 5.33 | -66 | -42 | -6 | 54% Middle Temporal Gyrus, posterior division, 29% Middle Temporal Gyrus, temporooccipital part |
| 92 | 4.99 | -16 | 64 | 18 | 82% Frontal Pole |
| 78 | 4.47 | -42 | 52 | 2 | 88% Frontal Pole |
| 41 | 4.07 | -38 | -18 | 18 | 50% Central Opercular Cortex, 20% Insular Cortex, 5% Parietal Operculum Cortex |
| 38 | 4.38 | -24 | -50 | 18 | 1% Precuneous Cortex |
| 34 | 4.16 | -64 | -8 | -14 | 44% Middle Temporal Gyrus, anterior division, 24% Middle Temporal Gyrus, posterior division, 8% Superior Temporal Gyrus, posterior division |

**Table 1.** Activation clusters from main effects of temperature and distraction in the *pooled cohort* obtained with cluster-forming threshold Z>3.09 and cluster-corrected p<0.05.

| Voxels | Z-MAX | X (mm) | Y (mm) | Z (mm) | Atlas labels |
| --- | --- | --- | --- | --- | --- |
| 9467 | 6.36 | 32 | -28 | 64 | 40% Postcentral Gyrus, 26% Precentral Gyrus |
| 3621 | 8.37 | 40 | -18 | 18 | 46% Central Opercular Cortex, 27% Parietal Operculum Cortex, 5% Insular Cortex |
| 3481 | 7.78 | -56 | -2 | 8 | 45% Central Opercular Cortex, 28% Precentral Gyrus, 5% Planum Polare |
| 1693 | 5.93 | -26 | 46 | 26 | 79% Frontal Pole |
| 287 | 4.98 | -62 | -56 | -10 | 54% Middle Temporal Gyrus, temporooccipital part, 21% Inferior Temporal Gyrus, temporooccipital part, 7% Lateral Occipital Cortex, inferior division |
| 285 | 5.2 | 4 | -26 | 8 | 28.2% Right Thalamus |
| 120 | 4.42 | 20 | -8 | 28 | 3.1% Right Caudate |
| 95 | 4.18 | 42 | 48 | 8 | 80% Frontal Pole |
| 86 | 4.71 | 16 | -20 | 10 | 099.8% Right Thalamus |
| 71 | 5.79 | -26 | -50 | -50 | 57% Left VIIIa, 35% Left VIIIb |
| 59 | 4.41 | 46 | 26 | 36 | 65% Middle Frontal Gyrus |
| 46 | 3.82 | -20 | -28 | 68 | 39% Precentral Gyrus, 23% Postcentral Gyrus |
| 38 | 4.17 | -42 | -72 | 24 | 72% Lateral Occipital Cortex, superior division |
| 37 | 4.11 | -18 | -38 | 66 | 51% Postcentral Gyrus |
| 36 | 3.84 | 2 | -66 | -38 | 69% Vermis VIIIa, 13% Vermis VIIIb |
| 34 | 3.83 | 24 | 54 | 26 | 76% Frontal Pole |
| 33 | 4.48 | 52 | 30 | 22 | 32% Middle Frontal Gyrus, 30% Inferior Frontal Gyrus, pars triangularis |
| 31 | 3.78 | 28 | 58 | 0 | 76% Frontal Pole |
| 1637 | 6.16 | 46 | -64 | 2 | 56% Lateral Occipital Cortex, inferior division, 10% Middle Temporal Gyrus, temporooccipital part |
| 1184 | 5.36 | -42 | -74 | 0 | 63% Lateral Occipital Cortex, inferior division |
| 176 | 5.38 | -26 | -76 | 30 | 67% Lateral Occipital Cortex, superior division |
| 126 | 4.73 | 26 | -52 | 50 | 34% Superior Parietal Lobule, 3% Lateral Occipital Cortex, superior division |
| 51 | 4.32 | -54 | -16 | 52 | 57% Postcentral Gyrus, 8% Precentral Gyrus |

**Table 2:** Results from intrasubject parametric regression with pain ratings in the *pooled cohort* obtained with cluster-forming threshold Z>3.09 and cluster-corrected p<0.05.

**Table 3.**

| **Parameter** | **Parameter estimate mean (SD)** | **Task modulation mean (SD)** | **Temp modulation mean (SD)** |
| --- | --- | --- | --- |
| *Group Mean* |  |  |  |
| ACC-PAG connection | 0.084 (0.0012) | 0.029 (0.0052) | - |
| ACC-LC connection | 0.058 (0.0011) | -0.019 (0.0054) | - |
| PAG-ACC connection | 0.083 (0.0012) | -0.0058 (0.0026) | - |
| PAG-RVM connection | 0.039 (0.0011) | -0.038 (0.0061) | - |
| LC-ACC connection | 0.054 (0.0012) | -0.012 (0.0052) | -0.0404 (0.0065) |
| RVM-PAG connection | 0.034 (0.0012) | -0.0004 (0.0026) | -0.0419 (0.0061) |

**Table 3.** Summary of mean parameter estimates for connections, and for task and temperature modulation in DCM.
